# Biomechanical analysis of sheep oesophagus subjected to biaxial testing including hyperelastic constitutive model fitting

**DOI:** 10.1016/j.heliyon.2022.e09312

**Published:** 2022-05-05

**Authors:** Harry Ngwangwa, Thanyani Pandelani, Makhosasana Msibi, Israel Mabuda, Letlhogonolo Semakane, Fulufhelo Nemavhola

**Affiliations:** a Unisa Biomedical Engineering Research Group, Department of Mechanical Engineering, School of Engineering, College of Science Engineering and Technology, University of South Africa, Pretoria, 0001, South Africa; b Defence and Security, Council for Scientific and Industrial Research (CSIR), Pretoria, 0001, South Africa

**Keywords:** Hyperelastic constitutive model, Soft tissue mechanics, Biomechanical properties of oesophagus

## Abstract

High quality computational model of soft tissues is a function of accurate and reliable mechanical properties. Hyperelastic constitutive models are normally utilised in developing reliable computational models. Therefore, section of proper and reliable constitutive models for soft tissue is critical. This work presents the biomechanical properties of oesophagus subjected to biaxial mechanical tensile test. Additionally, six hyperelastic constitutive models commonly used for modelling behaviour of soft tissues were selected. The experimental data were then fitted on Fung, Choi-Vito, Holzapfel (2000), Holzapfel (2005), Polynomial (Anisotropic) and Four-Fiber Family hyperelastic constitutive models. The sheep oesophagus subjected to equi-biaxial tension has exhibited different stress magnitude in both longitudinal and circumferential directions. There is significant difference between circumferential and longitudinal stresses (p = 0.0034). The average circumferential and longitudinal stresses are recorded to be 82.87 ± 30.36 kPa and 41.42 ± 32.02 kPa, respectively (p = 0.0034). Between six hyperelastic constitutive models, it was observed that Four-Fiber model has produced better fit when compared to others. After fitting biaxial mechanical properties of oesophagus, it was found that the Four-fiber family hyperelastic constitutive model would best fit.

## 1 Introduction

The oesophagus is usually defined as the hollow long pipe that connects the mouth from the pharynx to the stomach. The main purpose of the oesophagus is mechanical transportation of food. Transportation of food is performed or achieved by a powerful coordinated muscle contraction that follows each other by impelling swallowed food [1]. Biomechanical understanding plays an important role in the understanding of various mechanisms of disease and may be utilised to fast track the development and implementation of therapies [2, 3, 4, 5, 6, 7]. Additionally, detailed understanding of complex structure and soft tissue mechanics is critical in the development of replacement tissues based on tissue engineered materials [8, 9]. Similarly, mechanical response of soft tissues plays a vital role in the development of accurate and reliable computational models [10, 11, 12]. Without reliable materials parameters obtained by fitting the experimental data, the accuracy of computational models may be questioned. Finite Element analysis has been used for number of decades to simulate the behaviour of biological tissues under mechanical strain [13, 14]. There are number of diseases associated with oesophagus including Oesophageal atresia (EA), Achalasia, Oesophageal cancer, Gastroparesis, Peptic Ulcer Disease, Swallowing Disorders [15, 16, 17, 18, 19, 20]. Number of studies in soft tissue experimental has be commissioned to study the behaviour of tissues subjected to mechanical forces [14, 21, 22, 23]. It has been proven that most soft tissues exhibit highly non-linear stress-strain behaviour when subjected to mechanical strain [24, 25].

Studying the mechanics of oesophagus remains critical in understanding various disease mechanisms. Tissue engineering development of oesophagus remains primary requirement for the management of long-gap oesophageal atresia [26]. The death rate related to the oesophageal atresia remains high at 4.6 % [27]. Oesophageal atresia (EA) is a rare abnormality with an occurrence of 1 in every single 2500–4500 births [26, 27, 28, 29]. As an example, it has been reported that nearly 10 % of children under the age of 11 years are affected by a long-gap oesophageal atresia (LGEA) [30].

While finite element model and computational models have been developed and utilised in studying the behaviour of mechanical behaviour of biological tissues, the outstanding challenge is the selection of accurate hyperelastic constitutive models. This work presents six hyperelastic constitutive models fitted in the equi-biaxial tensile experimental data. The intention is to select the best hyperelastic constitutive model that may be utilised in numerical simulation of the sheep oesophagus subjected to equi-biaxial tensile forces.

## 2 Materials and methods

### 2.1 Tissue acquisition and preparation

**Figure 1:**
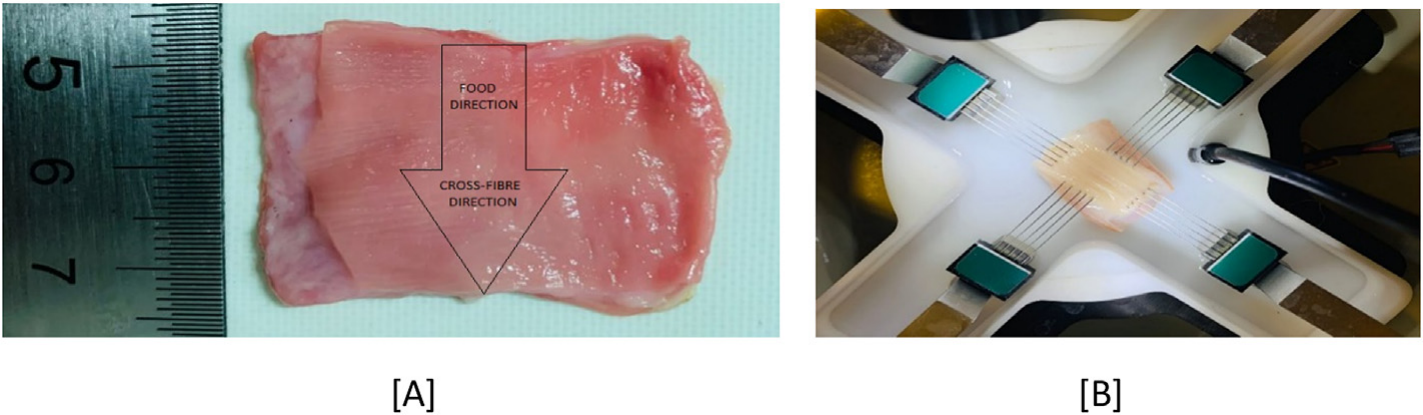
Experimental set-up of biaxial testing of sheep oesophagus. (A) shows the 20 × 40 mm oesophagus sample. (B) shows the BioTester system used for biaxial testing of sheep oesophagus including the rake assembly for clamping and water bath for mimicking the body temperature.

Thoracic organs of Vleis merino (40–42 kg) sheep breed were collected from a local abattoir and all sent to the Unisa biomedical laboratories for research purposes. The oesophagus was dissected from the sheep digestive organs for detailed soft tissue mechanical research experiments. One fresh sheep oesophagus was then sliced into 13 equal samples longitudinally and then opened circumferentially for testing. The food direction was marked as the longitudinal direction (0^o^) and its perpendicular direction was denoted as the circumferential direction. For all the samples, a full cross-section of 20 × 40 mm was dissected out of the extracted oesophagus by first dissecting 20 mm equally along the longitude and then 40 mm across the circumference (Figure 1A). The mechanical tests were then conducted immediately upon receiving the samples. All the samples are then marked numerically and kept soaked into a saline solution to sustain freshness throughout the experimental tests.

### 2.2 Biaxial mechanical testing

CellScale Biaxial testing system was used to capture the mechanical properties of all tissue samples. All prepared tissue samples were mounted in the custom biaxial tensile material testing apparatus (BioTester 5000 CellScalle, Wateroo, ON, Canada®) specifically designed for soft tissue mechanical testing. The BioTester 5000 biaxial system is installed with a unique system that uses rakes for piercing the through the tissue. In this test, the four rakes (see Figure 1B) are utilised to clamp the mucosa (soft inner) and submucosa (thick outer) tissue sample for biaxial tensile testing. 13 sliced equal samples of the sheep oesophagus were subjected to equi-biaxial tensile testing. The major dimensions such as length, width and the thickness of each sample were measured using a steel ruler and to ensure accuracy, all the measurements are double checked by a vernier calliper. Before collecting data, the 20 cycles precondition was conducted by applying a 10 % strain on the sample at a strain rate of 0.667/s. A preload of 5 mN was applied on each sample. To maintain hydration and mimicking the body temperature, saline 0.91% w/v of NaCl was placed in the bath and heated to 37 °C (maximum temperature of the heater scale) and maintained for the duration of testing. Each sample was subjected to 1.667/s equi-biaxial strain rate (50% strain for 30 s). 50 % strain was selected to be the biological peristalsis to represent expansion magnitude of the sheep oesophagus [31].

### 2.3 Tissue stress-strain analysis

**Figure 2:**
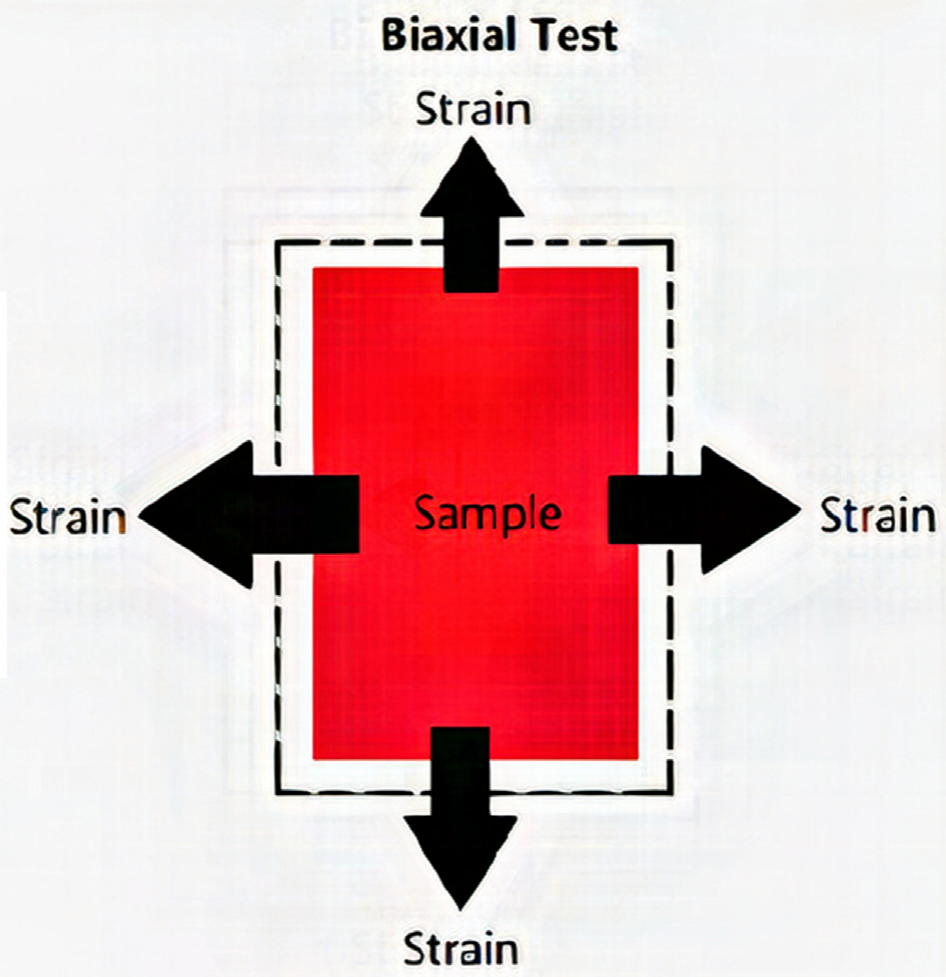
Stress-strain direction undeformed and under different strain rate.

In this study the stresses were calculated through the first Piola-Kirchoff stress P in the two-directions using the equation:

(1) $$P_{ii}=\frac{F_{ii}}{A_{0}}=\frac{F_{ii}}{L_{i0}h_{0}}$$

where F is the applied force in direction i = 1,2 and for the current study these indices represent longitudinal (cross-fibre-direction) and circumferential (fibre-direction) direction (see Figure 2).

The $A_{0}$ denotes the undeformed area with L representing the tissue length, and h the tissue thickness.

The infinitesimal strains were calculated by the formula:

(2) $$\varepsilon_{i}=\frac{\Delta L}{L_{0}}=\frac{L_{i}-L_{i0}}{L_{i0}}$$

The calculated stress results are cut-off at 50% strain. These stress results however are noisy therefore they were further filtered with an 8-point moving average filter in Excel. The data were resampled and further smoothened using different quadratic functions (Modelling). Later, the elastic modulus is used for significance tests in the linear region.

### 2.4 Hyperelastic constitutive modelling

The anistropic hyperelasticity formulation was considered to model the tissue. Hence, the anistropic hyperelastic constitutive models were deployed to define the relationship between the stain state and strain energy function. All the shear components together with the stress and strain in radial direction were neglected. The considered hyperelastic models are described as:

o Fung Model

Regardless of a minimum number of material parameters required, Fung model gives a fairly accurate stress-strain relationship measured from experiment [32].

(3) $$W=\frac{c}{2\;}\left(e^{Q}-1\right)$$

Differentiating the strain energy with respect to corresponding Green strains gives the Kirchhoff stresses as:

(4) $$S_{\theta \theta }=\frac{\partial W}{\partial E_{\theta \theta }}=c\left(a_{1}E_{\theta \theta }+a_{4}E_{zz}\right)e^{Q}$$

(5) $$S_{zz}=\frac{\partial W}{\partial E_{zz}}=c\left(a_{2}E_{zz}+a_{4}E_{\theta \theta }\right)e^{Q}$$

$c,\;and\;a_{i}$ are the material parameters.

Kirchhoff stress in circumferential direction ($S_{\theta \theta }$) is a nonlinear function of Green strain in circumferential direction ($E_{\theta \theta }$) only when the longitudinal strain in the longitudinal direction, $E_{ZZ}=0$. This is the similar situation in the axial direction.

Parameters are easily interpreted with reference to overall anisotropy and stiffness on both the Fung and Choi-Vito models [32].

o Choi-Vito model

The choi-Vito model and Fung model are similar. However, the Choi-Vito model is more advantageous because it has the terms for the different directions in separate exponentials [32].

The Choi-Vito strain energy function is given by [33]:

(6) $$W=b_{0}\left[exp\left(b_{1}E_{\theta \theta }^{2}\right)+exp\left(b_{2}E_{zz}^{2}\right)+exp\left(2b_{3}E_{\theta \theta }E_{zz}\right)-3\right]$$

Differentiating the strain energy with respect to corresponding Green strains gives the Kirchhoff stresses, circumferential and longitudinal, respectively as:

(7) $$S_{\theta \theta }=\frac{\partial W}{\partial E_{\theta \theta }}=b_{0}\left(2b_{1}E_{\theta \theta .}e^{b_{1}E_{\theta \theta }^{2}}+2b_{3}E_{zz\;}e^{2b_{3}E_{\theta \theta }E_{zz}}\right)$$

(8) $$S_{zz}=\frac{\partial W}{\partial E_{zz}}=b_{0}\left(2b_{2}E_{zz.}e^{b_{2}E_{zz}^{2}}+2b_{3}E_{\theta \theta }e^{2b_{3}E_{\theta \theta }E_{zz}}\right)$$

where $b^{\prime }s$ are the material constants.

o Four Fiber Models

In the four fiber model, the tissue is assumed to consist of an isotropic solid with embedded structural fibers. The fibers are oriented in four (4) different directions (one axial, one circumferential, and 2 diagonal directions) [33]. All the constitutive models considered in this study are summarised in Table 1.

**Table 1:** Strain energy functions of Fung, Choi-Vito, Holzapfel (2000), Holzapfel (2005), Polynomial (Anisotropic) and Four-Fiber Family hyperelastic constitutive models were fitted in the experimental data.

Model No.	Model	Strain Energy Function (SEF)	References
1	Fung constitutive model	$W=\frac{c}{2}\left(e^{Q}-1\right)$ Where $Q=b_{1}E_{\theta \theta }^{2}+b_{2}E_{ZZ}^{2}+b_{3}E_{RR}^{2}+2b_{4}E_{\theta \theta }E_{ZZ}+2b_{5}E_{ZZ}E_{RR}+2b_{6}E_{RR}E_{\theta \theta }$; and $b_{i}$ are the material parameters. The model is implemented in a polynomial format.	[34]
2	Choi-Vito model	$W=b_{0}\left[exp\left(b_{1}E_{11}^{2}\right)+exp\left(b_{2}E_{22}^{2}\right)+exp\left(2b_{3}E_{11}E_{22}\right)-3\right]$ Where $b_{i}$ are the material parameters. The model is implemented in an exponential format	[35]
3	Holzapfel (2000) model	$W=\frac{c_{1}}{2c_{2}}\left[exp\left(c_{2}{\left(I_{4}-1\right)}^{2}\right)-1\right]$ Where $c_{i}$ are the material parameters. The model is implemented in an exponential format.	[36]
4	Holzapfel (2005) model	$W=\frac{c_{1}}{2c_{2}}\left\{exp\left[c_{2}\left(\left(1-\kappa \right){\left(I_{1}-3\right)}^{2}+\kappa {\left(I_{4}-1\right)}^{2}\right)-1\right]\right\}$ Where $c_{i}$ are the material parameters and κ is a parameter that modulates the convergence rate.	[37]
5	Four-fiber family model	$W=\frac{c}{2}\left(I_{1}-3\right)+\overset{4}{\underset{i=1}{\sum }}\frac{c_{1i}}{4c_{2i}}\left\{exp\left[c_{2i}{\left(I_{4i}-1\right)}^{2}\right]-1\right\}\;$ This model implements a hybrid polynomial and exponential format where $c,\;c_{1i},c_{2i}$ are material parameters	[38, 39]
6	Polynomial (Anisotropy) model	$W=\overset{3}{\underset{i=1}{\sum }}a_{i}{\left(I_{1}-3\right)}^{i}+\overset{3}{\underset{j=1}{\sum }}b_{j}{\left(I_{2}-3\right)}^{j}+\overset{6}{\underset{k=2}{\sum }}c_{k}{\left(I_{4}-1\right)}^{k}+\overset{6}{\underset{m=2}{\sum }}e_{m}{\left(I_{6}-1\right)}^{m}$ Where $a_{i},b_{j},c_{k},\;and\;e_{m}$ are material parameters.	[40]

### 2.5 Data analysis

In this study, the constrained optimisation by linear approximation algorithm (COBYLA (3^rd^ party: SciPy)) implemented in Hyperfit software was used in fitting the equi-biaxial tensile experimental data of Fung, Choi-Vito, Holzapfel (2000), Holzapfel (2005), Polynomial (Anisotropic) and Four-Fiber Family hyperelastic constitutive models. A number of important metrics are used to measure the models’ fitting accuracies.

Initially the coefficient of determination, R^2^, (also known as Nash-Sutcliffe coefficient) is defined as follows:

(9) $$R^{2}=1-\frac{\sum_{i=1}^{n}{\left(y_{e}-y_{m}\right)}^{2}}{\sum_{i=1}^{n}{\left(y_{e}-\underline{y_{e}}\right)}^{2}}$$

where $y_{e}$ is the experimental data, $y_{m}$ is the model predicted data, $y_{e}^{=}$ is the average value of the experimental data, the indices i,…,n denote the data points, and $R^{2}\varepsilon \left\langle -\infty ,1\right\rangle$, where a perfect fit is defined for $R^{2}=1$.

From R^2^, we define the Evaluation Index, which is a critical parameter in evaluating how the hyperelastic constitutive model fits the experimental data. This index evaluates how a model compares from one experimental data set to another set. The EI was previously defined as follows [41]:

(10) $$Evaluation\;Index\;\left(EI\right)=\left[\frac{R-R_{minimum}}{R_{maximum}-R_{minimum}}\right]$$

where,

(11) $$R=abs\left[\left(1-R^{2}\right)\;\right]$$

where *R* is defined as the quantity that is dependent on R^2^ and is expressed in Eq. (11). The *R*_*minimum*_ *and R*_*maximum*_ represent the *R* values for poorest and best fitting hyperelastic models, respectively. EI in Eq. (10) is a comparative parameter whose values may span values between 0.0 for poorest fitting models, and 1.0 for best fitting models. Therefore, the higher the coefficient of determination (R^2^), the higher the model fit (EI).

Another important metric is the correlation coefficient (r), which may be define as

(12) $$r=\frac{\sum_{i=1}^{n}\left(y_{e}-\underline{y_{e}}\right)\left(y_{m}-\underline{y_{m}}\right)}{\sqrt{\sum_{i=1}^{n}{\left(y_{m}-\underline{y_{m}}\right)}^{2}.\sum_{i=1}^{n}{\left(y_{m}-\underline{y_{m}}\right)}^{2}}}$$

where $\underline{y_{m}}$ is an average value of the model predicted data, and all the other quantities in Eq. (12) are defined as given in Eq. (9).

The module results presented in Section 3 also report the values of the Normalized Root Mean Square error (NRMSE) defined as follows:

(13) $$NRMSE=\frac{\sqrt{\frac{1}{n}\sum_{i=1}^{n}{\left(y_{e}-y_{m}\right)}^{2}}}{abs\left(\underline{y_{e}}\right)}$$

From a Normalised error (NE) may also be defined as follows:

(14) $$NE=\frac{\frac{1}{n}\sum_{i=1}^{n}abs\left(y_{e}-y_{m}\right)}{abs\left(\underline{y_{e}}\right)}$$

## 3 Experimental results

**Figure 3:**
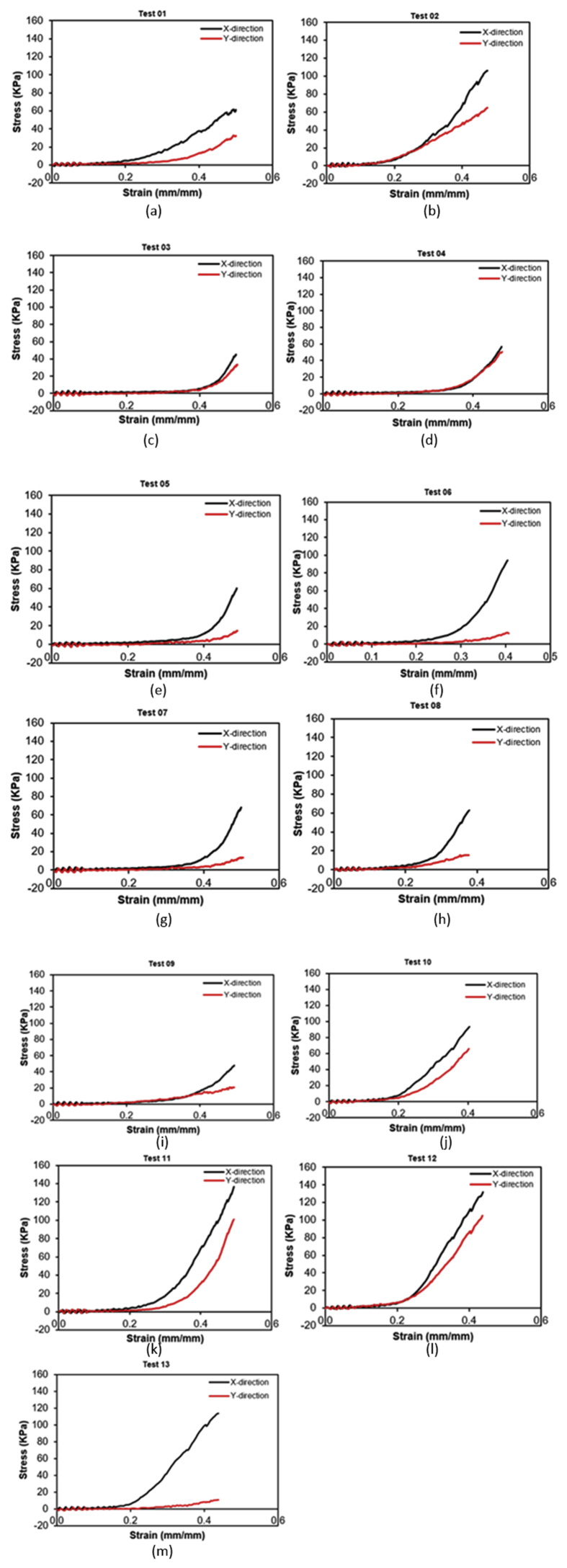
Experimental engineering stress and strain tensile data of sheep oesophugus (N = 13) subjected to equi-biaxial mechanical test 1 (a) to test 13 (m). X and Y directions representing, circumferential and longitudinal directions, respectively.

In this study, thirteen specimens of sheep oesophagi were subjected to the equi-biaxial tensile test. The limitation of hook slipping was minimised and specimens that showed this behaviour were discarded and excluded from the test results. The force and displacement data obtained during testing were then converted to engineering stress and strain using Eqs. (1) and (2), respectively (Figure 3). It was clearly observed that the oesophagus soft tissue is anisotropic and as such exhibit different mechanical properties depending on the direction in which the force was applied. To understand the stresses, two directions were defined. The direction along the length of the oesophagus was termed the longitudinal direction, while the perpendicular direction to the longitudinal was termed circumferential direction. The stresses in the radial direction were safely neglected due to the large ratios between the tissue thickness and lengths of the specimen in the longitudinal and circumferential directions and, due to the fact that the tissues were only loaded in the plane.

Figure 3 shows the stress vs strain data for the sheep oesophagus soft tissue subjected to equi-biaxial tensile force. It can be clearly seen that the stress and strain trend exhibit anisotropic behaviour. Also, the graphs clearly indicate that the sheep oesophagus soft tissue is highly non-linear in its mechanical behaviour. The circumferential direction shows much higher engineering stress than that in the longitudinal direction. This could be so because the circumferential direction is the direction that has more fibers. This is beneficial to the physiological functioning of the oesophagus muscle in propelling swallowed food into the stomach where peristaltic contractions are excited along this direction of the oesophageal body. In this study, the fiber direction was not precisely determined and as such, only two directions based on the physics of the oesophagus were defined.

**Figure 4:**
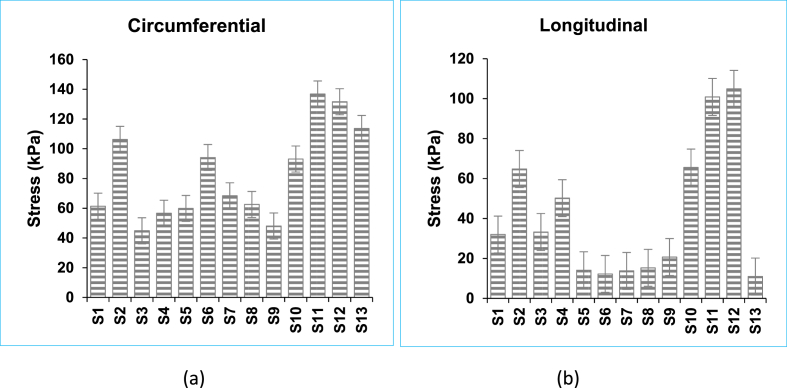
Stress with standard error values taken at maximum strain for each specimen. The average (a) circumferential and (b) longitudinal stresses are recorded to be 82.87 ± 30.36 kPa and 41.42 ± 32.02 kPa, respectively.

Figure 4 shows the stress at 45% strain for both circumferential and longitudinal directions. It can be clearly seen that the circumferential direction is on average twice as much as stress in the longitudinal direction. The average circumferential and longitudinal stresses are recorded to be 82.87 ± 30.36 kPa and 41.42 ± 32.02 kPa, respectively. A test of significance in the differences between the average values of the stresses in these two directions yielded a p-value equal to 0.0034, which actually shows very high significance.

**Figure 5:**
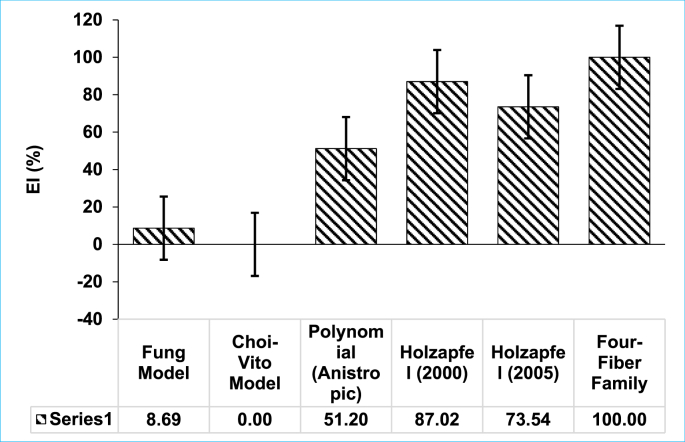
Evaluation Index (EI) with standard error calculated from the average coefficient of determination (R^2^) based on the fitting of the hyperelatic constitituve models.

One of the objectives of this study was to select sizable number of commonly used hyperelastic constitutive models and fit the equi-biaxial tensile experimental data. This was done to ensure that the material parameters of each constitutive model are then recorded for further use in the area of computational model development. Therefore, six (6) hyperelastic constitutive models were selected, namely, Fung, Choi-Vito, Holzapfel (2000), Holzapfel (2005), Polynomial (Anisotropic) and Four-Fiber Family. Furthermore, these constitutive models were then compared to each other using the coefficient of determination (R^2^). Table 1 shows the mathematical relationship and the strain energy functions of the Fung, Choi-Vito, Holzapfel (2000), Holzapfel (2005), Polynomial (Anisotropic) and Four-Fiber Family hyperelastic constitutive model. Each coefficient of determination for equivalent sample and constitutive model was then presented in Tables 2, 3, 4, 5, 6, and 7 . It must also be noted that in addition to the coefficient of determination (R^2^), three other parameters were added in this to measure the efficiency of fitted hyperelastic model, namely, Correlation coefficient (r), Normalised Root Mean Square Error (NRMSE), and Normalised Error (NE), whose expressions are presented in Eqs. (12), (13), and (14), respectively. Evaluation Index (EI) was also used as a means to evaluate the efficiency and effectiveness of different hyperelastic constitutive models as shown in Eq. (5). Figure 5 shows the Evaluation Index (EI) of all hyperelastic constitutive models considered in this study. It was found that the average EI for Fung, Choi-Vito, Holzapfel (2000), Holzapfel (2005), Polynomial (Anisotropic) and Four-Fiber Family constitutive models are 8.69 %, 0.00 %, 87.02 %, 73.54 %, 51.20 % and 100 %, respectively (Figure 5). From the data presented on EI, the best fit was produced by Four-Fiber family constitutive model followed by the Holzapfel (2000) model. The worse constitutive model fit was Choi-Vito as it has an Evaluate Index of 0.00 % (See Figure 5).

**Table 2:** Fung hyperelastic constitutive model fitted on the equi-biaxial tensile experimental data to evlauate the six term material parameters (i.e c, b_1_, b_2_, b_3_, b_4_, b_5_ and b_6_) including Coefficient of Determination (R^2^), Correlation Coefficient (r), Normalised Error (NE) and Norm. RMS Error (NRMSE).

	S1	S2	S3	S4	S5	S6	S7	S8	S9	S10	S11	S12	S13	Ave	STD
c	2.00	2.00	0.12	0.36	0.14	0.52	0.65	1.26	2.00	2.00	2.00	2.00	2.00	1.31	0.79
b1	1.13	1.26	4.18	3.92	2.10	3.87	2.87	3.73	0.86	1.77	1.19	1.96	2.00	2.37	1.15
b2	-0.11	0.71	1.89	2.18	2.07	-1.07	0.41	0.50	-0.18	-0.25	-0.37	0.08	-0.10	0.44	0.98
b3	0.69	1.74	-3.60	-3.65	-1.34	1.01	-1.09	-0.32	0.66	1.03	0.73	1.47	1.50	-0.09	1.76
b4	0.95	0.38	0.93	1.12	1.43	1.20	1.37	2.08	0.93	1.06	1.57	0.95	0.63	1.12	0.41
b5	-0.15	-1.31	-3.30	-4.25	2.68	0.73	0.96	0.43	-0.20	-0.95	-0.43	-0.40	-0.30	-0.50	1.70
b6	-2.11	-2.51	-0.12	1.51	-6.33	-6.07	-2.49	-2.63	-1.88	-3.71	-2.42	-3.70	-4.19	-2.82	2.04
2.00	0.87	0.88	0.97	0.88	0.97	0.59	0.97	0.95	0.84	0.82	0.86	0.64	0.86	0.85	0.11
r	0.98	0.96	1.00	0.99	0.99	0.99	0.99	0.98	0.98	0.98	0.99	0.96	0.97	0.98	0.01
NRMSE	0.32	0.30	0.22	0.45	0.20	0.62	0.16	0.22	0.33	0.46	0.41	0.60	0.41	0.36	0.14
NE	0.24	0.23	0.15	0.26	0.16	0.42	0.14	0.17	0.28	0.31	0.26	0.41	0.35	0.26	0.09

**Table 3:** Choi-Vito hyperelastic constitutive model fitted on the equi-biaxial tensile experimental data to evlauate the six term material parameters (i.e c, b_1_, b_2_ and b_3_) including Coefficient of Determination (R^2^), Correlation Coefficient (r), Normalised Error (NE) and Norm. RMS Error (NRMSE).

	S1	S2	S3	S4	S5	S6	S7	S8	S9	S10	S11	S12	S13	Ave	STD
c	4.07	12.24	0.06	0.47	2.11	4.54	10.07	15.00	15.00	4.22	3.34	7.15	0.66	6.07	5.15
b1	1.08	1.59	3.97	2.65	2.55	4.50	1.12	2.93	0.48	3.21	2.45	2.00	0.32	2.22	1.23
b2	1.20	0.77	5.23	1.10	-1.93	-3.19	-1.22	0.56	-0.60	2.49	1.95	2.24	-0.65	0.61	2.10
b3	3.25	2.76	10.63	8.11	3.71	4.44	1.72	0.60	1.28	6.17	5.21	5.06	0.74	4.13	2.84
2	0.52	0.92	0.96	0.99	0.08	1.64	0.25	0.80	0.90	0.90	0.89	0.93	0.87	0.82	0.36
r	0.99	0.99	1.00	1.00	0.96	0.97	0.95	0.94	0.97	0.99	0.99	0.99	0.98	0.98	0.02
NRMSE	0.60	0.24	0.26	0.13	1.22	1.71	0.86	0.52	0.29	0.35	0.36	0.29	1.33	0.63	0.48
NE	0.50	0.18	0.17	0.10	0.91	1.23	0.71	0.45	0.25	0.30	0.30	0.23	1.08	0.49	0.36

**Table 4:** Polynomial (Anisotropic) hyperelastic constitutive model fitted on the equi-biaxial tensile experimental data to evlauate the six term material parameters (i.e *a*_*1*_*, a*_*2*_*, a*_*3*_*, b*_*1*_*, b*_*2*_*, b*_*3*_*, c*_*2*_*, c*_*3*_*, c*_*4*_*, c*_*5*_*, c*_*6*_, ​φ) including Coefficient of Determination (R^2^), Correlation Coefficient (r), Normalised Error (NE) and Norm. RMS Error (NRMSE).

	S1	S2	S3	S4	S5	S6	S7	S8	S9	S10	S11	S12	S13	Ave	STD
a1	0.23	2.00	0.20	2.30	0.10	0.24	0.25	0.37	1.82	0.49	0.41	1.11	6.71	1.25	1.74
a2	1.99	7.91	-2.43	-1.26	3.24	-0.09	-7.13	1.12	-0.53	3.14	-7.58	1.19	25.33	1.92	7.84
a3	0.42	-0.02	-1.43	1.86	7.57	0.35	6.69	1.27	0.99	4.15	7.12	5.78	-8.72	2.00	4.26
b1	4.57	0.10	4.37	5.14	-9.28	0.35	-6.82	1.87	3.30	2.59	10.09	1.37	-0.36	1.33	4.81
b2	4.77	1.70	0.09	1.74	15.19	0.33	-2.58	1.80	1.64	5.92	7.78	2.02	17.70	4.47	5.72
b3	-4.62	3.41	7.07	2.32	10.69	2.82	9.04	1.67	3.11	-2.85	10.05	0.94	8.15	3.98	4.60
c2	-1.90	-0.54	1.53	-0.47	6.56	1.43	6.29	0.32	0.02	-1.24	0.04	-4.00	-4.43	0.28	3.13
c3	3.06	-0.64	1.83	-1.21	15.99	-2.56	-6.33	0.23	-0.55	2.03	2.79	3.94	15.92	2.65	6.24
c4	-0.41	2.56	-1.34	0.06	3.58	-0.35	1.00	-1.61	-0.54	1.72	1.36	-1.34	14.12	1.45	3.96
c5	0.66	-0.81	0.52	-0.37	-5.21	4.40	-1.24	1.47	1.22	2.50	-1.81	13.96	18.02	2.56	6.18
c6	-0.48	0.19	1.27	1.22	2.07	0.82	0.18	2.24	-0.20	-2.91	0.37	-9.71	13.32	0.64	4.71
phi	0.16	0.00	0.76	0.73	2.31	0.30	2.13	-0.03	0.06	0.24	0.32	0.54	-0.70	0.52	0.81
2.00	0.97	1.00	0.99	1.00	0.71	0.98	0.88	0.99	0.99	1.00	0.99	1.00	0.78	0.94	0.09
r	0.99	1.00	0.99	1.00	0.92	0.99	0.94	0.99	0.99	1.00	1.00	1.00	0.89	0.98	0.03
NRMSE	0.14	0.05	0.14	0.08	0.62	0.15	0.33	0.11	0.10	0.07	0.08	0.05	0.43	0.18	0.17
NE	0.12	0.04	0.12	0.06	0.52	0.10	0.27	0.08	0.08	0.06	0.07	0.04	0.34	0.15	0.14

**Table 5:** Holzapfel (2000) hyperelastic constitutive model fitted on the equi-biaxial tensile experimental data to evlauate the six term material parameters (i.e *(*μ,*k*_*1*_*, k*_*2*_*and*φ) including Coefficient of Determination (R^2^), Correlation Coefficient (r), Normalised Error (NE) and Norm. RMS Error (NRMSE).

	S1	S2	S3	S4	S5	S6	S7	S8	S9	S10	S11	S12	S13	Ave	STD
mu	11.66	19.30	0.20	0.50	0.80	0.53	0.78	0.61	1.90	0.50	0.50	0.50	0.50	2.95	5.57
k1	11.11	22.19	0.18	0.95	0.24	1.66	0.44	2.04	1.02	8.68	5.66	12.20	7.62	5.69	6.31
k2	0.27	0.30	2.63	2.03	2.56	2.67	2.07	2.67	1.46	1.38	1.18	1.11	0.95	1.64	0.83
phi	0.66	-0.71	0.71	0.77	0.44	-0.34	0.42	-0.49	0.61	-0.68	-0.68	-0.72	-0.27	-0.02	0.60
2.00	0.98	0.98	0.99	0.99	0.98	0.98	0.99	0.96	0.96	0.98	0.98	0.97	0.94	0.97	0.01
r	0.99	0.99	1.00	1.00	0.99	0.99	0.99	0.98	0.98	0.99	0.99	0.99	0.98	0.99	0.01
NRMSE	0.11	0.13	0.10	0.13	0.16	0.19	0.12	0.20	0.17	0.17	0.17	0.19	0.28	0.16	0.05
NE	0.09	0.11	0.08	0.11	0.12	0.14	0.09	0.16	0.14	0.15	0.15	0.17	0.24	0.13	0.04

**Table 6:** Holzapfel (2005) hyperelastic constitutive model fitted on the equi-biaxial tensile experimental data to evlauate the six term material parameters (i.e *(*μ,*k*_*1*_*, k*_*2*_*,*φ,andρ) including Coefficient of Determination (R^2^), Correlation Coefficient (r), Normalised Error (NE) and Norm. RMS Error (NRMSE).

	S1	S2	S3	S4	S5	S6	S7	S8	S9	S10	S11	S12	S13	Ave	STD
mu	0.10	0.50	0.07	0.50	0.40	0.50	0.39	0.27	1.04	0.50	0.50	0.50	0.50	0.44	0.23
k1	4.33	8.83	0.20	0.94	0.24	1.57	0.44	2.05	1.01	8.50	0.70	11.12	7.42	3.64	3.77
k2	0.28	0.21	2.31	0.84	2.56	2.72	2.07	2.68	1.46	0.36	0.65	0.26	0.96	1.34	0.96
phi	0.00	0.00	0.70	0.00	-0.44	-0.33	-0.42	-0.50	-0.60	1.56	0.00	3.03	0.25	0.25	0.98
rho	0.58	0.36	0.92	0.07	1.00	1.00	1.00	1.00	1.00	0.00	0.07	0.00	1.00	0.62	0.43
2.00	0.98	0.98	0.99	0.99	0.98	0.97	0.99	0.96	0.96	0.89	0.99	0.95	0.92	0.97	0.03
r	0.99	0.99	1.00	0.99	0.99	0.99	0.99	0.98	0.98	1.00	1.00	0.99	0.98	0.99	0.01
NRMSE	0.12	0.13	0.10	0.14	0.16	0.20	0.12	0.20	0.17	0.36	0.11	0.25	0.30	0.18	0.08
NE	0.11	0.10	0.08	0.11	0.12	0.16	0.09	0.16	0.14	0.29	0.10	0.20	0.27	0.15	0.06

**Table 7:** Four-Fiber Family hyperelastic constitutive model fitted on the equi-biaxial tensile experimental data to evlauate the six term material parameters (i.e *(c, c*_*11*_*, c*_*21*_*, c*_*12*_*, c*_*22*_*, c*_*134*_*, c*_*234*_*and*$\varphi_{0}$) including Coefficient of Determination (R^2^), Correlation Coefficient (r), Normalised Error (NE) and Norm. RMS Error (NRMSE).

	S1	S2	S3	S4	S5	S6	S7	S8	S9	S10	S11	S12	S13	Ave	STD
c	0.00	0.56	0.44	0.03	0.47	0.50	0.29	0.50	0.01	0.01	1.90	0.50	0.50	0.44	0.47
c1_1	1.65	0.32	0.62	2.16	0.30	0.13	0.62	0.00	4.49	0.01	0.07	0.43	0.00	0.83	1.23
c2_1	0.04	0.10	1.78	1.79	1.86	1.59	0.75	0.00	0.95	0.06	0.00	0.01	0.00	0.69	0.77
c1_2	15.25	14.50	0.65	0.00	0.62	4.30	0.00	5.99	1.55	21.64	16.01	21.32	15.61	9.03	8.14
c2_2	0.52	0.91	1.21	0.33	1.66	2.67	2.76	2.75	1.79	1.22	1.05	1.06	0.91	1.45	0.79
c1_34	1.15	14.37	0.05	1.05	0.25	1.15	0.90	1.92	1.38	6.60	1.84	13.39	7.40	3.96	4.76
c2_34	1.27	0.46	3.60	2.11	2.81	2.68	2.03	1.60	0.56	1.62	1.81	1.16	1.00	1.75	0.87
phi0	-1.46	-1.01	-0.54	0.35	-0.31	-0.58	-0.37	1.57	0.64	-1.93	-1.61	-1.04	-0.38	-0.51	0.92
2.00	0.99	0.98	0.99	0.99	0.98	0.98	0.99	0.98	0.99	0.98	0.99	0.97	0.94	0.98	0.01
r	0.99	0.99	1.00	1.00	0.99	0.99	0.99	0.99	1.00	0.99	0.99	0.99	0.98	0.99	0.00
NRMSE	0.10	0.12	0.11	0.12	0.15	0.19	0.11	0.15	0.10	0.16	0.13	0.19	0.27	0.14	0.05
NE	0.09	0.10	0.09	0.10	0.12	0.14	0.08	0.12	0.08	0.14	0.11	0.17	0.24	0.12	0.04

**Figure 6:**
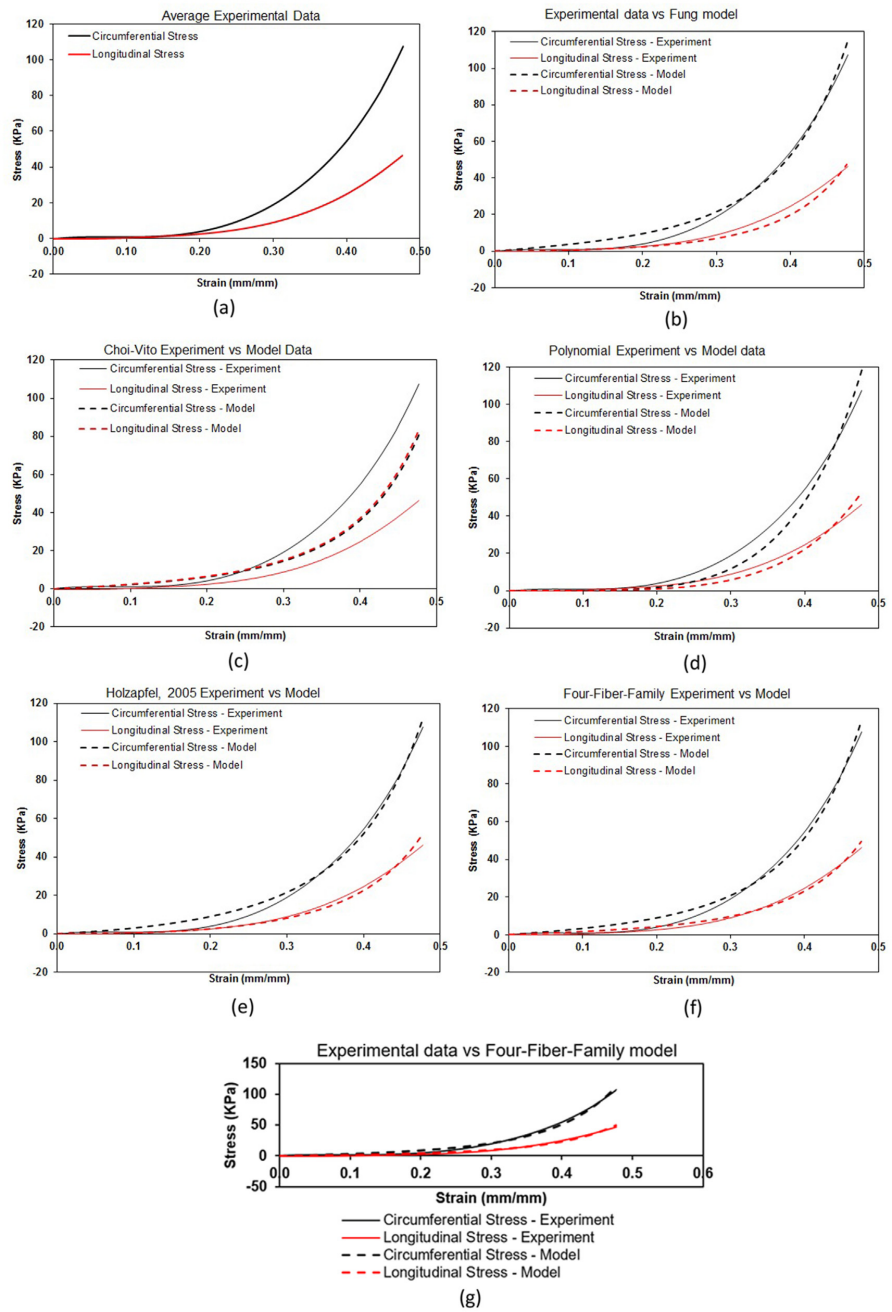
(a) Mean experimental engineering stress and strain tensile data of sheep oesophugus subjected to equi-biaxial mechanical test. Constitutive parameters fitted simultaneously to the averaged responses of 13 specimens for (b) Fung, (c) Choi-Vito, (d) Polynomial (anisotropic), (e) Holzapfel (2000),(f) Holzapfel (2005) and (g) Four-Fibre-Family hyperelastic models.

The experimental stress and strain averages were determined and compared with the six constitutive models as shown in Figure 6. The solid lines represent the experimental stresses (x and y direction) and the dotted lines represent the model stresses results. It is evident from the Figures below that there is less error between the experimental results and models results. For all the models (Fung, Choi-Vito, Polynomial, Holzapel 2000, Holzapel 2005, and Four-Fiber Family), both maximum stresses (longitudinal and circumferential) are attained at the strain of 0.48 mm/mm for all the models.

## 4 Discussion

Understanding the mechanical properties of the oesophagus is vital for the development of the computational model to provide more insight into physics related to the oesophagus. To achieve that goal, this paper focuses on and presents material parameters of six hyperelastic constitutive models. Most of the previous studies report on material parameters calculated using the Fung constitutive model. It is known that many biological soft tissues display nonlinear behaviour and have viscoelastic characteristics. They are almost incompressible if not incompressible and they experience large deformations [31]. In this study, the rectangular shaped specimen was applied with the load in two perpendicular directions, namely circumferential and longitudinal directions by means of hooks anchored on each side. Biaxial testing was conducted on arbitrarily locations of the oesophagus. Thirteen (13) specimens were prepared and tested using biaxial test equipment set to 50 % displacement of specimen to stretch the tissues. The strain rate was chosen based on the functionality (expansion and contraction) or physiological conditions of the sheep oesophagus, more than 50 % strain was considered unrealistic or close to impossible.

Moreover, the study of determining the rat oesophagus shear modulus and its dependence on the longitudinal and circumferential stresses and strains, the oesophagus exhibited the anisotropic behaviour at the stretched state. The oesophagus was found to be more stiffer in the longitudinal direction than in the circumferential direction [42]. The biaxial mechanical testing to assess the mechanical behaviour of the porcine oesophagus was performed. Six 16 mm square specimen with a thickness of 3 mm were analysed utilizing a biaxial testing system in a bath with water at 37 °C. A single 40 % load was applied in both directions, longitudinal and circumferential. The oesophageal tissue exhibited minor anisotropic behaviour with stress of 4 MPa and 3 MPa in the circumferential axis and longitudinal axis, respectively at 40% strain. Additionally, elastic modulus was found to be 1.6 kPa at 40 % strain in the circumferential axis and 1.3 kPa at 40 % strain in the longitudinal axis [43].

In this study, the biomechanical properties, and the material parameters of selected hyperelastic constitutive models were reported. While there have been various studies in determining the mechanical properties of oesophagus, this study presents the biomechanical properties of sheep oesophagus that have been conducted without separating layers. In addition, in this study, we have presented six (6) hyperelastic constitutive models that may be further utilised for the development of computational models. In our understanding, there is no study that has presented six possible hyperelastic constitutive models for sheep oesophagus. In addition, previously presented results on mechanics of oesophagus have presented data on uniaxial tensile testing [44]. Animal models like rabbit [45], rat [44, 46, 47], pig, sheep have been previously utilised for studying the mechanics of oesophagus ex-vivo. The oesophagus tissue has four layers namely, mucosa, submucosa, muscularis propria, and adventitia. In this study, the equi-biaxial force was applied in the whole layer of the oesophagus tissue. This approach is in contrast with various studies where the forces were applied to the sub-layers [44, 48, 49, 50] instead of applying the mechanical load on the whole layer. The present study is very important because it helps understand the overall mechanical behaviour of the oesophagus muscle.

The stress-strain curves for the various directions (circumferential and longitudinal) of the specimen were generated from the biaxial tests results. Based on this study, the mechanical behaviour of sheep oesophagus is non-linear and anisotropic. The tissue properties vary between the two directions, circumferential and longitudinal. However, the oesophagus exhibits isotropic properties between 0 and 0.2 mm/mm strain for all the specimen, having the same stiffness in the x and y directions. The modulus of elasticity is constant and equal in both directions at this strain range. Moreover, the oesophagus exhibits nearly pronounced isotropic properties for specimens 03 and 04, which may point to the fact that the organisation of biological soft tissue is highly random and may vary widely from one region to another within the same organ of the same host.

As compared to what has been presented in this paper, there are some researchers who used more simplified constitutive models like Ogden in analysing the mechanical behaviour of soft tissues [11]. Additionally, mechanical behaviour of materials like soft polymers have utilised constitutive models in fitting the mechanical experimental data [51]. The results presented here could be utilised in the development of more effective biomaterials and finite element modelling of soft tissues. Previously, it has been reported that conditions like myocardial infarction could be studied by developing accurate and reliable computational models by utilising material parameters obtained from constitutive models like Fung [13, 52, 53].

According to the stress-strain graphs, the oesophagus exhibits higher stresses along the x-axis than in the y-axis. This implies that, the oesophagus has more strength in the x-axis which may be attributed to the functionality of the oesophagus and the direction of the fibers. However, the orientation of the fibres themselves was not studied in this paper.

All soft tissues exhibit common mechanical properties on some features. They are not truly elastic, they exhibit pseudo-elastic behaviours [54, 55, 56]. Some researchers [44, 57, 58] have analysed the oesophagus by considering three layers, mucosa, submucosa and the muscle separately. In addition, the multiaxial mechanical behaviour of the ovine (resembling sheep) oesophagus was investigated [58]. The biaxial tensile test was one of the performed tests and the ovine oesophagus exhibits a heterogeneous and anisotropic behaviour with various mechanical properties for each layer (mucosa-submucosa and muscle layer). Modelling of Oesophagus study, the two layers(mucosa-submucosa and muscles) were analysed as elastic shells, each layer having its own zero-stress state, and elastic constants [44]. The properties in each layer were determined from the pressure-diameter relation and zero-stress state. The submucosa layer was found to be the stiffer [44].

The six constitutive models (Fung, Choi-Vito, Holzapfel (2005), Holzapfel (2000), Four-Fiber Family) and Polynomial) were considered to fit the experimental data. The Four-Fiber Family proved to fit the experimental data well when compared to other models, this is evident from the results plotted in Figure 5 which are further supported by the results in Tables 2, 3, 4, 5, 6, and 7 whose average values for the R^2^, r, NRMSE, and NE are plotted in Figure 6. Although the EI results in Figure 5 show a wider margin in performance between the two Holzapfel models, the plot of the average errors and correlations in Figure 6 do not really show any significant differences. Overall, it would look like a combination of the absence of the φ parameter in both the Choi-Vito and Fung models may have a negative effect on their performance. However, the mode material parameters in Fung model affords it a slight advantage over the Choi-Vito. From a computational point of view, it is remarkable how a material model with very few material parameters such as the Holzapfel (2000) can rival the performance of a material model that has almost twice as many material parameters as itself. Hence, the two Holzapfel models may prove to fit the experimental data well. These two models could be considered for the development of the computational model.

### 4.1 Limitations of study

One of the limitations of this study is that the four different layers that are normally seen in the oesophageal tissue are treated as one solid tissue. This may pose challenges especially during mechanical pulling on the tissue because if not treated well, there may be relative slipping of tissue layers. While this study provided equi-biaxial tensile data, it is planned that future studies should implement tri-axial data including the imaging of sample while under tension.

Other limitations of this study include the following:

o Image processing was not done to determine the direction of the fiber
o Only 6 constitutive models were considered
o The oesophagus was not classified in terms of the segments (cervical, thoracic and abdominal.
o The specimens were kept at 37 ° C instead of 39° C (sheep body temperature) due to maximum temperature of the heater scale.

## 5 Conclusion and significance of research

This study aims at understanding the mechanical properties of the sheep oesophagus in both longitudinal and circumferential directions through examination of the performances of six different constitutive hyperelastic material models. The tensile testing was conducted along these two perpendicular directions neglecting the radial direction due to its relative size. It is understood that this may introduce some minor errors especially in the calculation of the stresses from the measured results. Despite the limitations given above, the study yields very important results in understanding the overall mechanical behaviour of the oesophagus muscle.

The following can be concluded as the important findings of this study on the sheep oesophagus:

o The oesophagus muscle is stronger in the circumferential direction which is deemed to be beneficial for its physiological function during peristaltic contraction when swallowed food is propelled into the stomach. It is also hypothesised that this physical activity may naturally cause more deposition of collagen and other fibrous material which result in making it stronger.
o The experiment exhibits anisotropic behaviour for higher strains and isotropic behaviour for small strains.
o The two Holzapfel models and Four-Fibre Family hyperelastic constitutive model are the best fit and could be considered for the development of computational model.

There are other factors such as the significance of the φ material parameter and the implicit modelling of the anisotropic strain invariants with an exponential framework that may require very closer examination in further studies. These are two very distinct features shared by two Holzapfel models and the Four-fibre family model.

## Declarations

### Author contribution statement

Harry Ngwangwa, Thanyani Pandelani, Makhosasana Msibi, Israel Mabuda, Letlhogonolo Semakane, Fulufhelo Nemavhola: Conceived and designed the experiments; Performed the experiments; Analyzed and interpreted the data; Contributed reagents, materials, analysis tools or data; Wrote the paper.

### Funding statement

Fulufhelo Nemavhola was supported by the 10.13039/501100001321National Research Foundation of South Africa (129380) and ASDG-RSP (00012).

### Data availability statement

Data included in article/supplementary material/referenced in article.

### Declaration of interests statement

The authors declare no conflict of interest.

### Additional information

No additional information is available for this paper.
